# New record of the lace bug species *Acalypta marginata* (Wolff) (Hemiptera: Heteroptera: Tingidae) from Japan

**DOI:** 10.3897/BDJ.9.e62868

**Published:** 2021-02-25

**Authors:** Jun Souma

**Affiliations:** 1 Entomological Laboratory, Graduate School of Bioresource and Bioenvironmental Sciences, Kyushu University, Fukuoka, Japan Entomological Laboratory, Graduate School of Bioresource and Bioenvironmental Sciences, Kyushu University Fukuoka Japan; 2 Research Fellowship for Young Scientists (DC1), Japan Society for the Promotion of Science, Tokyo, Japan Research Fellowship for Young Scientists (DC1), Japan Society for the Promotion of Science Tokyo Japan

**Keywords:** Heteroptera, Tingidae, *Acalypta marginata*, lace bug, new record, key to species, Japan, Hokkaido

## Abstract

**Background:**

The lace bug species *Acalypta marginata* (Wolff, 1804) has, to date, been widely known to occur in the Palaearctic Region, but has not been recorded from Japan.

**New information:**

*Acalypta marginata* is recorded from Japan for the first time. Its habitat in Japan is the grassland of Hokkaido. A key to the species of *Acalypta* occurring in Japan is provided.

## Introduction

The lace bug species *Acalypta marginata* (Wolff, 1804) (Hemiptera: Heteroptera: Tingidae) is widely distributed in the Palaearctic Region (Péricart and Golub 1996, Aukema et al. 2013). In eastern Asia, this lace bug has been known to occur in Korea and Mongolia to date (Golub 1982, Cho et al. 2020).

In Japan, seven species of the genus *Acalypta* Westwood, 1840, namely: *A. cooleyi* Drake, 1917, *A. gracilis* (Fieber, 1844), *A. hirashimai* Takeya, 1962, *A. miyamotoi* Takeya, 1962, *A. pallidicoronata* Souma, 2019, *A. sauteri* Drake, 1942 and *A. tsurugisana* Tomokuni, 1972 have been recorded to date (Yamada and Ishikawa 2016, Souma 2019, Souma 2020).

Recently, I sorted a collection of Heteroptera from Hokkaido, Japan and found an undetermined species of *Acalypta*. After careful morphological examination, I concluded that the undetermined species represents *A. marginata*. Here, I report *A. marginata* from Japan for the first time. Additionally, I provide a key to *Acalypta* species occurring in Japan.

## Materials and methods

Dried specimens were used. Morphological characteristics were observed under a stereoscopic microscope (SZ60, Olympus, Tokyo, Japan). Specimens were photographed using a digital microscope (VHX-1100, Keyence, Osaka, Japan). Distribution records of species were mapped using SimpleMappr (Shorthouse 2010). Geographical coordinates were obtained from Google Maps. The terminology used in this study generally follows that of Drake and Ruhoff (1965). All the specimens used in this study were deposited at the Laboratory of Entomology, Faculty of Agriculture, Tokyo University of Agriculture, Kanagawa, Japan (TUA).

## Taxon treatments

### Acalypta marginata

557B707A-18E4-50BD-AB13-614E38EB6CD5

Acanthia marginata : Wolff 1804: 132, new species, description and figures.
Acalypta marginata : Horváth 1906: 26, new combination, description; [Bibr B6510901][Bibr B6510870]: 169, monograph, description and figures; Golub 1982: 201, distribution and biology; Péricart 1983: 111, monograph, description and figures; Golub 1988: 140, key to species and figures; Péricart and Golub 1996: 8, catalogue; Rédei et al. 2004: 75, biology; Rintala and Rinne 2011: 74, monograph, description and figures; Aukema et al. 2013: 58, catalogue; Cho et al. 2020: 738, distribution and biology.

#### Materials

- **Type status:** Other material. **Occurrence:** recordedBy: Shigehisa Hori; individualCount: 3; sex: male; lifeStage: adult; **Location:** islandGroup: Japanese archipelago; island: Hokkaido; country: Japan; stateProvince: Hokkaido; municipality: Abashiri; locality: Oketo-chô, Nakayama-fûketsu; decimalLatitude: 43.58597226; decimalLongitude: 143.4748237; geodeticDatum: WGS84; **Identification:** identifiedBy: Jun Souma; dateIdentified: 2019; **Event:** samplingProtocol: none specified; eventDate: 1994-07-08–20; **Record Level:** institutionCode: TUA; basisOfRecord: PreservedSpecimen
- **Type status:** Other material. **Occurrence:** recordedBy: Shigehisa Hori; individualCount: 2; sex: male; lifeStage: adult; **Location:** islandGroup: Japanese archipelago; island: Hokkaido; country: Japan; stateProvince: Hokkaido; municipality: Shirataki-mura; locality: Mt. Hirayama; decimalLatitude: 43.77086032; decimalLongitude: 143.02416775; geodeticDatum: WGS84; **Identification:** identifiedBy: Jun Souma; dateIdentified: 2019; **Event:** samplingProtocol: pitfall trap near Japanese butterbur; eventDate: 1993-07-20; **Record Level:** institutionCode: TUA; basisOfRecord: PreservedSpecimen

#### Diagnosis

*Acalypta marginata* can be differentiated from other species of *Acalypta* by a combination of the following characteristics: both macropterous and brachypterous morphs known; body fuscous, elongate in brachypterous morph (Fig. 1a, b) and oblong in macropterous morph; antenniferous tubercles obtuse, slightly curved inwards; basal part of antennal segment III not thickened; rostrum reaching posterior margin of metasternum (Fig. 1c); pronotum tricarinate, 0.8 times as long as maximum width across paranota; hood concealing basal eighth of vertex; pronotal carinae laminate; calli coarsely punctate; paranotum with 2 rows of areolae at widest part; anterolateral angle of paranotum rounded, not protruding anteriad; posterolateral angle of paranotum not protruding posteriad; posterior process 2.2 times as wide as its length; posterior margin of hemelytron in brachypterous morph weakly sinuate; gap between both hemelytra in brachypterous morph distinctly narrower than discoidal area at widest part of each; costal area with a single row of areolae throughout its length; subcostal area with 3–5 rows of areolae at widest part; discoidal area with 3–5 rows at widest part, expanded beyond apical fourth of hemelytron in brachypterous morph, as wide as subcostal area at widest parts of each; and basal third of discoidal-sutural boundary vein carinate.

#### Distribution

**Figure 2.**
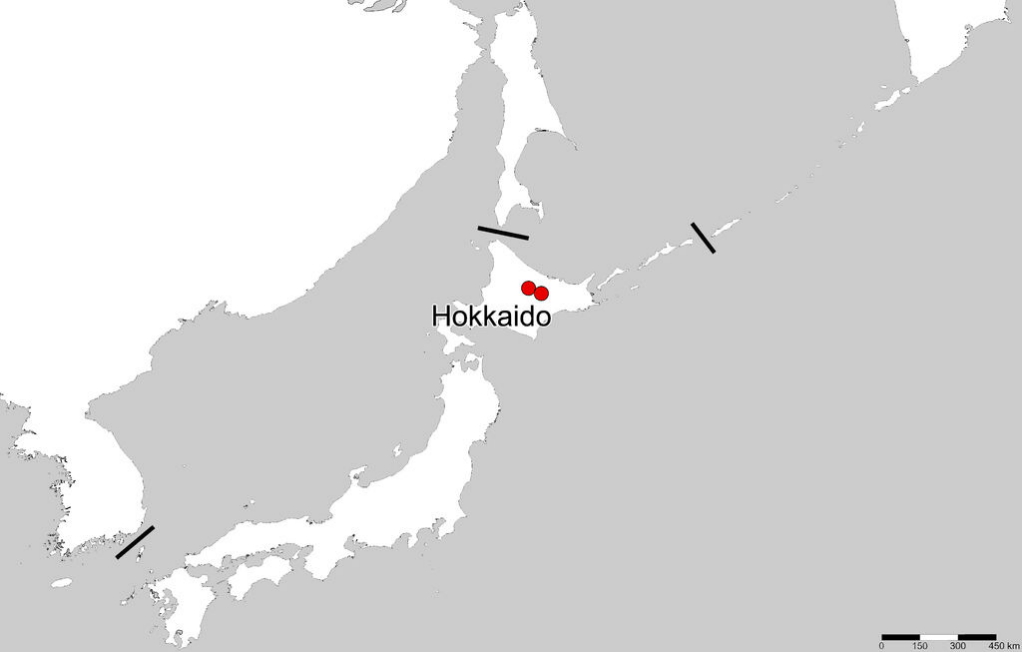
Collection sites for *Acalypta marginata* from Hokkaido, Japan.

Japan (Hokkaido) (Fig. 2), Armenia(?), Austria, Azerbaijan, Belgium, Bosnia Herzegovina, Bulgaria, Byelorussia, Croatia, Czech Republic, Finland, France, Germany, Hungary, Italy, Latvia, Luxembourg, Moldavia, Mongolia, Netherlands, Norway, Poland, Portugal, Romania, Russia, Serbia, Slovakia, Slovenia, Spain, Sweden, Switzerland, Turkey, Ukraine (Péricart and Golub 1996, Aukema et al. 2013, Cho et al. 2020).

#### Biology

The host plant for *Acalypta marginata* in Japan is unknown. In other distribution areas, *A. marginata* is found on *Rhytidiadelphus* sp. (Hylocomiaceae); it is also found on herbaceous plants (Putshkov 1974, Péricart 1983, Rédei et al. 2004, Rintala and Rinne 2011).

*Acalypta marginata* is found in grasslands in Japan. In other distribution areas, *A. marginata* is known to be found in dry and sunny environments, such as grassy ground; however, it is sometimes collected from humid and shaded environments, such as the forest floor (cf. Putshkov 1974, Rédei et al. 2004).

In Japan, adults have been collected only in July and the overwintering stage is unknown. In other distribution areas, adults are found in almost all seasons and the overwintering stage is known to be the adult or elder nymph (Putshkov 1974, Péricart 1983, Rédei et al. 2004, Rintala and Rinne 2011).

#### Taxon discussion

The above-recorded specimens match well with the photographs of the non-types (Rintala and Rinne 2011), descriptions (Wolff 1804, Péricart and Golub 1996) and keys (Putshkov 1974, Golub 1988) of *Acalypta marginata* described from Hungary, based on the brachypterous morph.

*Acalypta marginata* is very similar to *A. nigrina* Fallén, 1807 in the shape of the antenniferous tubercles and the pronotum; however, the former is distinguished from the latter by the following characteristics: body fuscous and elongate in brachypterous morph (Fig. 1a, b). In contrast, *A. nigrina* has the following features: body dark grey and ovate in brachypterous morph.

## Identification Keys

### Key to the species of *Acalypta* occurring in Japan

**Table:** 

1	Both macropterous and brachypterous morphs known; pronotum tricarinate; hypocostal lamina of hemelytron with a single row throughout its length	2
–	Only brachypterous morph known; pronotum unicarinate; hypocostal lamina of hemelytron with 2 rows of areolae in basal part and a single row in remaining parts	4
2	Antenniferous tubercles pointed at apices, straight; anterolateral angle of paranotum angular, strongly protruding anteriad, reaching mid-level of compound eye	*A. cooleyi* Drake, 1917
–	Antenniferous tubercles obtuse, slightly curved inwards; anterolateral angle of paranotum rounded, not protruding anteriad	3
3	Basal part of antennal segment III thickened	*A. gracilis* (Fieber, 1844)
–	Basal part of antennal segment III not thickened	*A. marginata* (Wolff, 1804)
4	Pronotum not less than 0.8 times as long as maximum width across paranota; discoidal area considerably expanded beyond apical fourth of hemelytron, distinctly wider than subcostal area	5
–	Pronotum not more than 0.6 times as long as maximum width across paranota; discoidal area not expanded beyond apical fourth of hemelytron, not wider than subcostal area	6
5	Posterolateral angle of paranotum protruding posteriad; posterior process 2.5 times as wide as its length; costal area with a single row of areolae throughout its length; subcostal area with 5 rows of areolae at widest part; discoidal area with 5 rows of areolae at widest part	*A. hirashimai* Takeya, 1962
–	Posterolateral angle of paranotum not protruding posteriad; posterior process 4 times as wide as its length; costal area with 2 rows of areolae in basal part and a single row in remaining parts; subcostal area with 7 rows of areolae at widest part; discoidal area with 6 rows of areolae at widest part	*A. pallidicoronata Souma*, 2019
6	Pronotum 0.6 times as long as maximum width across paranota; anterolateral angle of paranotum weakly protruding anteriad, not reaching mid-level of compound eye; posterolateral angle of paranotum protruding posteriad; costal area with 2 rows of areolae in basal part	*A. sauteri* Drake, 1942
–	Pronotum 0.5 times as long as maximum width across paranota; anterolateral angle of paranotum strongly protruding anteriad, reaching mid-level of compound eye; posterolateral angle of paranotum not protruding posteriad; costal area with 3–4 rows of areolae in basal part	7
7	Paranotum with 3 rows of areolae throughout its length; costal area with 3 rows of areolae in basal part, a single row in middle part and 2 rows in apical part; and discoidal area as wide as subcostal area at widest part of each	*A. miyamotoi* Takeya, 1962
–	Paranotum with 4 rows of areolae throughout its length; costal area with 4 rows of areolae in basal part, 2 rows in middle part and 3 rows in apical part; discoidal area narrower than subcostal area at widest part of each	*A. tsurugisana* Tomokuni, 1972

## Supplementary Material

XML Treatment for Acalypta marginata

## Figures and Tables

**Figure 1a.**
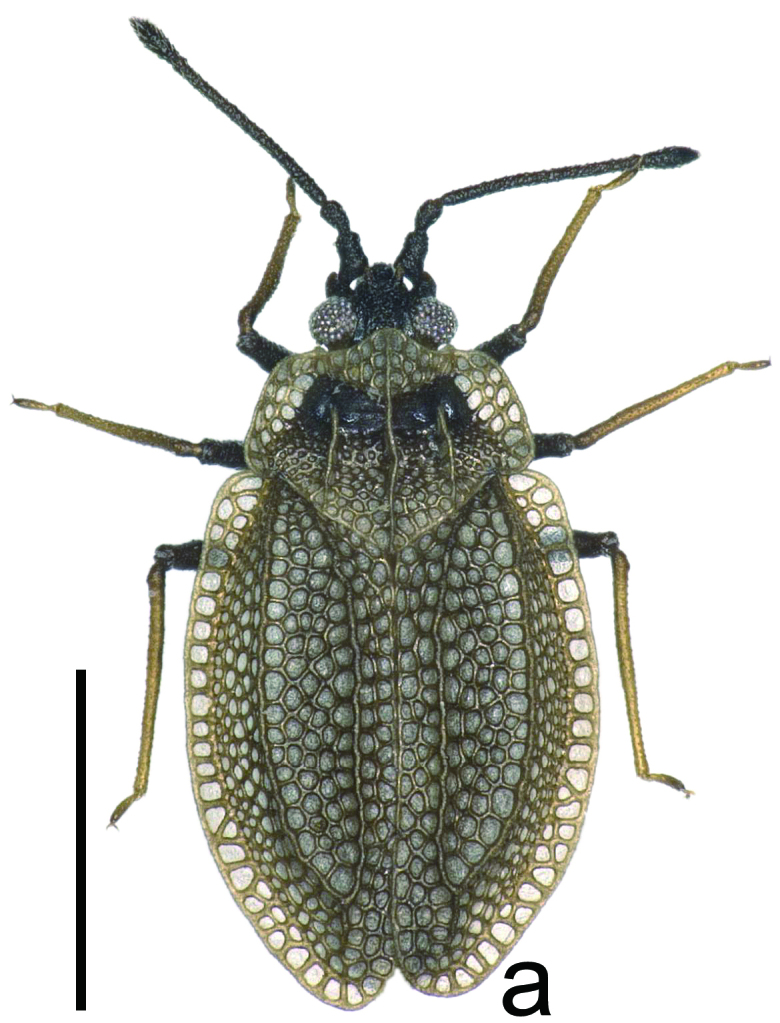
brachypterous male, dorsal view. Scale bar 1.0 mm.

**Figure 1b.**
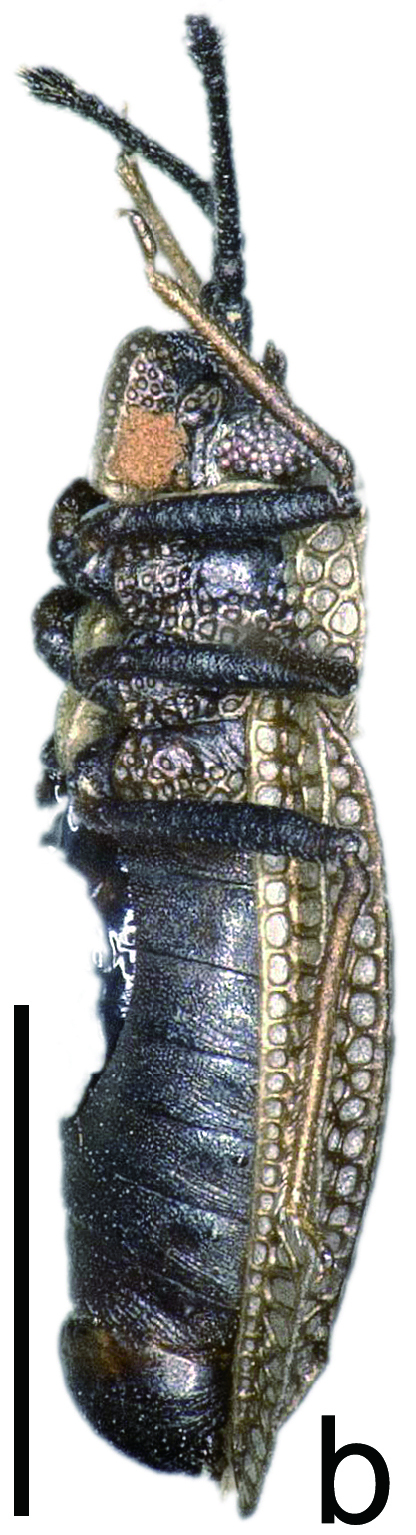
brachypterous male, lateral view. Scale bar 1.0 mm.

**Figure 1c.**
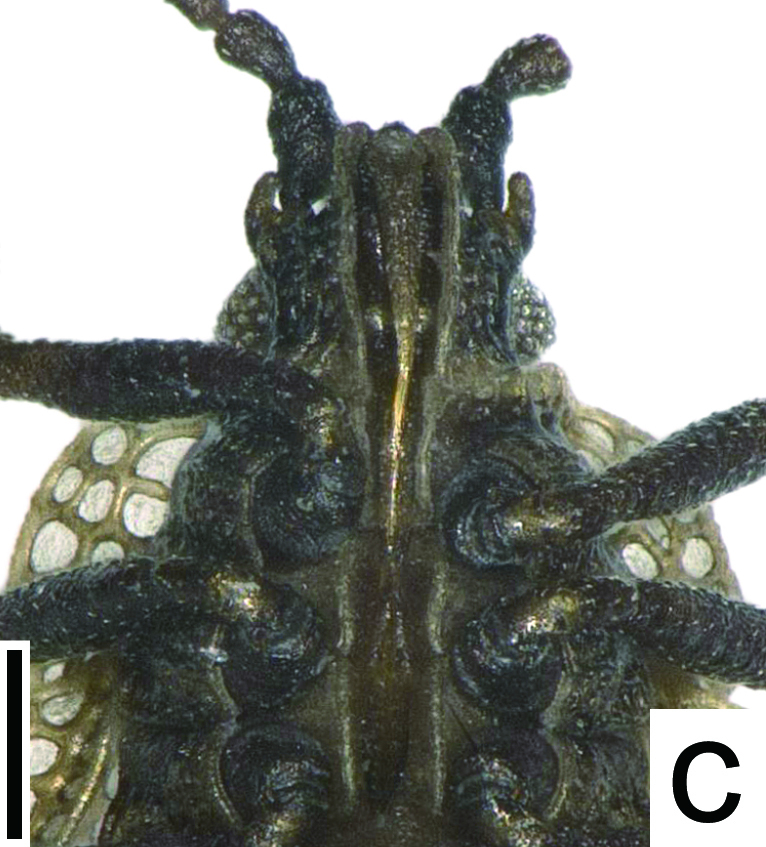
rostrum, ventral view. Scale bar 0.2 mm.
